# Biodegradable pins fixation for lateral condylar fracture of the humerus in children: A retrospective study of biodegradable pin fixation combined with versus without absorbable tension band wiring

**DOI:** 10.1097/MD.0000000000050972

**Published:** 2026-10-16

**Authors:** Lifei Dong, Dingding Jia, Liyuan Hao, Zhenyu Chen, Xiuyu Fan, Na Li

**Affiliations:** a Pediatric Orthopedic Ward, Xingtai City Orthopedics Hospital, Xingtai City, Hebei Province, China.

**Keywords:** biodegradable pins, children, lateral condylar fracture

## Abstract

Biodegradable pins are increasingly used in the treatment of lateral condylar fractures of the humerus (LCFH) in children, but the strength of the biodegradable pin is weaker than that of K-wire. However, to enhance the stability of LCFH fixed by biodegradable pins, absorbable tension band wiring is often combined with the application, yet high-quality comparative evidence verifying the clinical value of this auxiliary reinforcement remains lacking. This study aimed to compare and analyze whether auxiliary absorbable tension band wiring has clinical significance for Weiss type III LCFH and to clarify whether additional tension band fixation is clinically necessary in reducing postoperative complications and improving fracture stability. This study enrolled 96 children with Weiss type III LCFH admitted between January 2017 and September 2019. The patients were divided into 2 groups: A (fixed by 3 biodegradable pins alone, n = 45) and B (fixed by 3 biodegradable pins and combined with the application of absorbable figure-of-eight tension band wiring, n = 51). Preoperative, intraoperative, and postoperative indicators, including age, sex, operation time, intraoperative blood loss, fracture healing time, elbow functional recovery time, and postoperative complications, were compared between the 2 groups. Postoperative elbow function was assessed by using the Flynn grading system. All radiographic measurements and functional evaluations were performed by 2 calibrated orthopedic surgeons with excellent inter-rater reliability. Sample size calculations were performed before data collection to guarantee statistical validity. There were no significant differences in age, sex, injured side, preoperative fracture displacement, operation duration, intraoperative blood loss, fracture healing time, elbow functional recovery time, or Flynn functional grading (all *P* > .05). Complete fracture union was observed in all patients, and no adverse complications, such as wound infection, bone nonunion, osteonecrosis, or elbow deformity, occurred in either group. Absorbable tension band wiring reinforcement is not required after biodegradable pin fixation in the treatment of LCFH in children.

## 1. Introduction

Lateral condylar fracture of the humerus (LCFH) is a common elbow fracture in children, second only to supracondylar humeral fracture,^[1,2]^ which is common in children 4 to 6 years old. Common complications include bone nonunion and elbow valgus. LCFH is an intra-articular fracture; no displacement or displacement of <2 mm is treated with plaster fixation,^[3]^ and fracture displacements of >2 mm require early open reduction and internal fixation (ORIF),^[4]^ which is mainly a Kirschner wire (KW).

KWs left outside the skin are easy to cause needle tract infection, and remaining inside the skin requires secondary surgery. Biodegradable pins can avoid these defects,^[5]^ Xingtai City Orthopedics Hospital has been applying biodegradable pins to fix the LCFH since 2017 and achieved good results, but some doctors are worried about the stability of the biodegradable pins, so additional absorbable line tension band were usually applied. To date, no study has specifically compared the importance of applying absorbable line tension bands after open reduction of biodegradable pins fixation in the treatment of LCFH.

The objective of this study was to compare fracture healing time and postoperative elbow functional recovery between pediatric Weiss type III LCFH treated with biodegradable pins alone versus biodegradable pins combined with absorbable tension band wiring. We also aimed to verify whether additional absorbable tension band reinforcement brings clinical benefits in reducing postoperative complications and improving fracture stability. Here, we reviewed the records of children fixed with biodegradable pins alone or combined with an absorbable tension band wiring to assess whether the assisted absorbable tension band wiring is clinically significant for the stability and prognosis of fractures.

## 2. Materials and methods

This retrospective observational study was approved by the Xingtai Science and Technology Bureau (Project Approval Number: 2022 ZC269) in December 2022. Written informed consent was obtained from the legal guardians of all included pediatric patients before surgical intervention. All clinical procedures implemented in this study strictly complied with the ethical standards stipulated by the institutional research committee and the 1964 Helsinki Declaration and its subsequent revised versions.

This study retrospectively analyzed LCFH treated with biodegradable pin fixation with or without an absorbable line tension band at Xingtai Orthopaedic Hospital from January 2017 to January 2019.

### 2.1. Patients

The inclusion criteria were as follows: LCFHs treated with biodegradable pin fixation with or without absorbable line tension band fixation at Xingtai City Orthopedics Hospital, aged 2 to 12 years. The exclusion criteria were: patients aged > 12 years old; multiple fractures; patients with incomplete clinical data; open fractures; the duration from injury to surgery was >2 weeks; follow-up times were <3 months; patients with a Weiss classification of type I or type II. A total of 96 patients met the inclusion criteria, all of whom were classified as Weiss type III, and had good parental compliance.45 simple biodegradable pin fixation, classified as group A, of which 33 were boys and 12 girls, age (5.4 ± 2.1); 51 patients in group B, biodegradable pin fixation-assisted absorbable line tension band fixation, 35 boys and 16 girls, age (5.2 ± 2.3).

### 2.2. Surgical technique

Under general anesthesia, an incision approximately 5 cm long was made to expose the front of the elbow using the lateral approach of the elbow joint. Pay attention to avoid iatrogenic articular cartilage injury. Do not dissect the rear of the humeral lateral condyle in order to avoid damage to the blood supply of the trochlea humeri and capitellum. After exposing the joint, remove the joint congestion and clots, and directly reduce the fracture. After reduction, the lateral condyle of the humerus was fixed with three 1.5 or 2.0 mm diameter KWs (K-wires with a diameter of 1.5 or 2.0 mm were selected according to the age of the child and the size of the fracture block). After measuring their length, they were replaced with 3 self-reinforced biodegradable pins made of poly-l-lactic acid (PLLA). Patients with absorbable tension band fixation were drilled with a 2.0 mm KW approximately 3 cm in the proximal part of the fracture. A 1-0 absorbable tension band, which was a “figure of 8-band wiring” was applied to suture the lateral condylar cartilage of the humerus. After surgery, the elbow was fixed in flexion and forearm functional position by using a plaster slab. All patients underwent fixation for 6 weeks (Figs. 1 and 2), respectively. Six weeks after the operation, the plaster slab was removed, and elbow radiography was reexamined. Clinical fracture healing was confirmed if continuous bony callus bridged the fracture line and the fracture margin became blurred on X-ray films. The guardians instructed the children to perform independent active elbow functional exercises without passive forced manipulation. Outpatient follow-up and elbow function evaluations were performed at 2 weeks, 6 weeks, and 3 months postoperatively.

**Figure 1.**
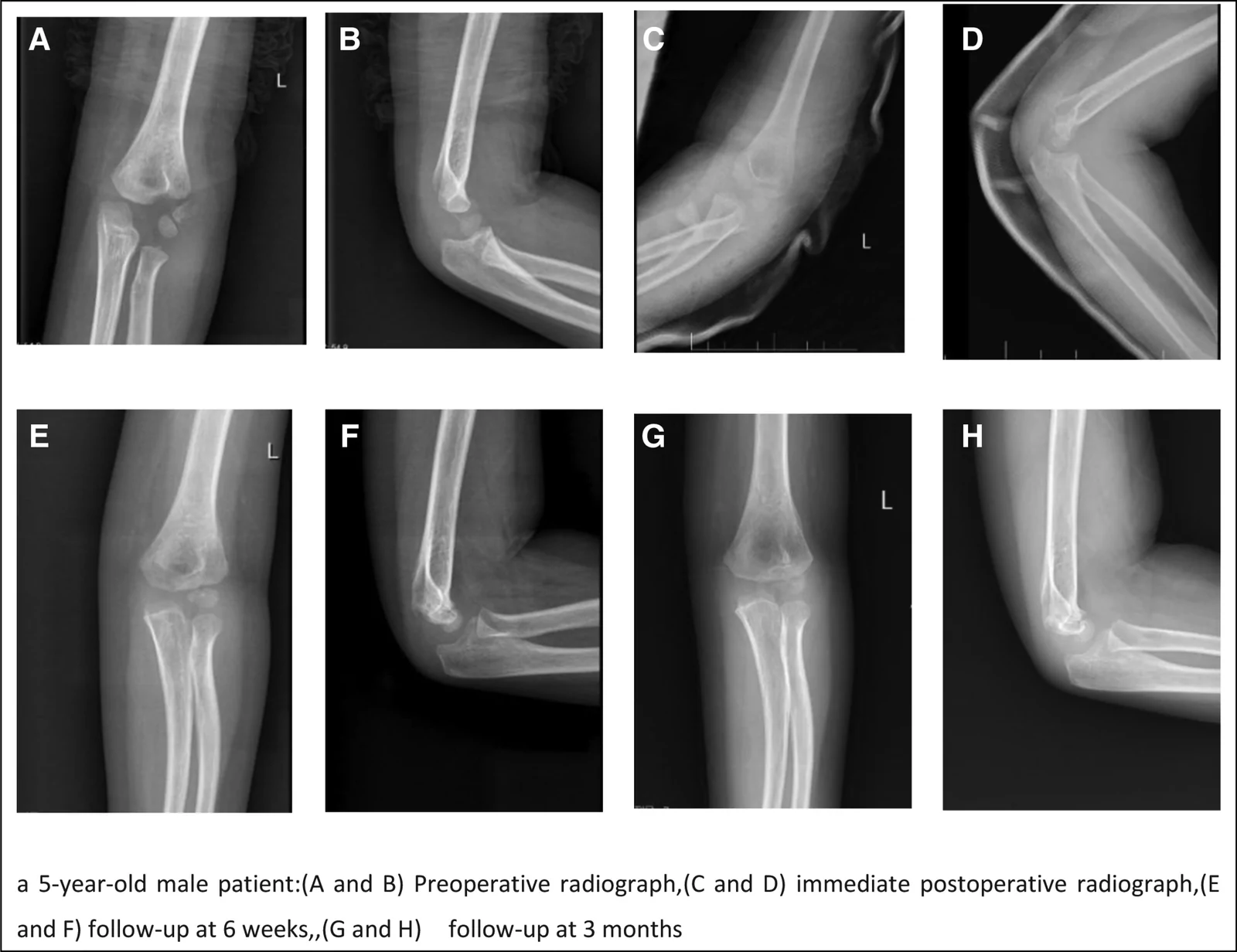
A 5-year-old male patient: (A and B) Preoperative radiograph, (C and D) immediate postoperative radiograph, (E and F) follow-up at 6 weeks, (G and H) follow-up at 3 months.

**Figure 2.**
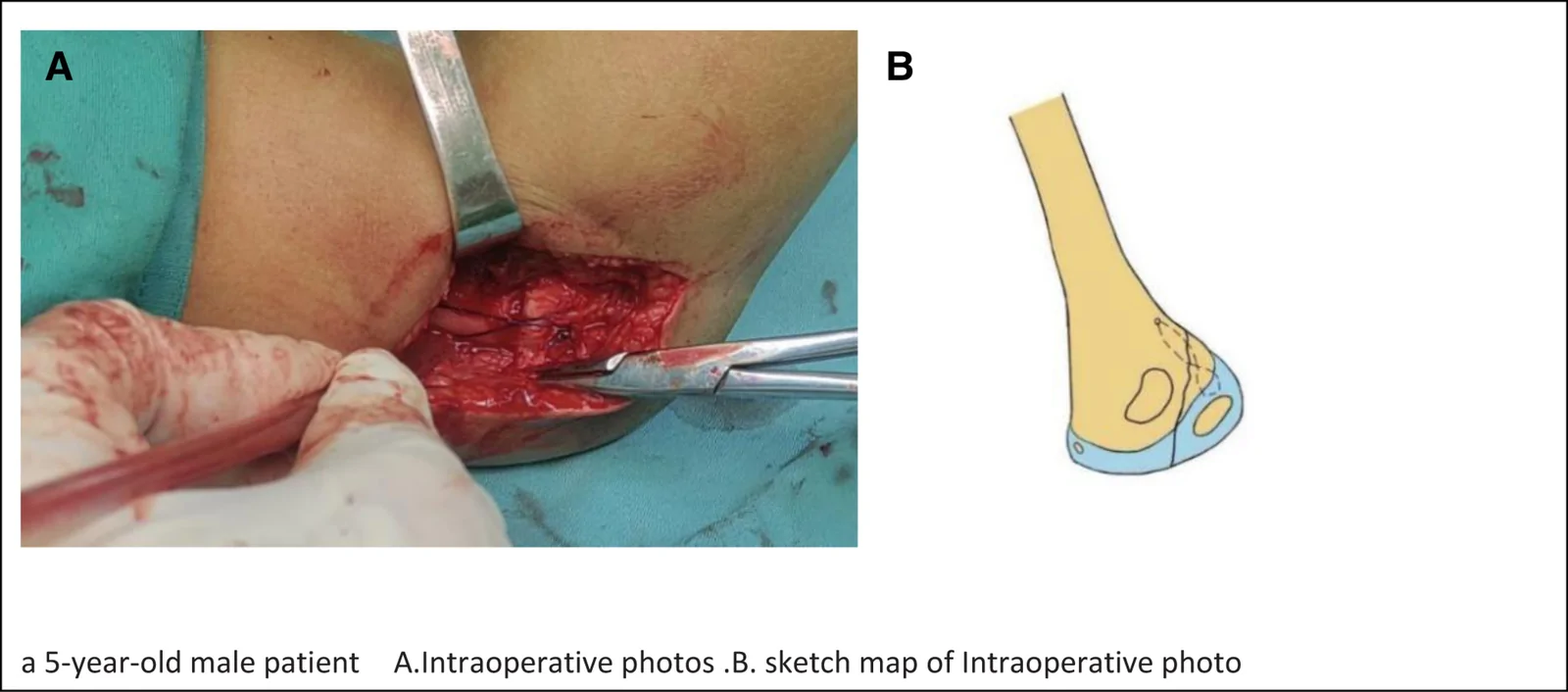
A 5-year-old male patient. (A) Intraoperative photos. (B) Sketch map of intraoperative photo.

### 2.3. Measurement assessment

All radiographic measurements and postoperative Flynn elbow function grading were independently completed by 2 attending orthopedic surgeons with over 5 years of specialized pediatric orthopedic clinical experience, who were blinded to the grouping information of each patient.

Before formal evaluation, unified training and calibration were conducted using 20 randomly selected radiographs excluded from this cohort to standardize the measurement and scoring criteria. Inter-rater reliability was quantified by Kappa consistency test: the Kappa value for preoperative fracture displacement measurement was 0.86, and the Kappa value for Flynn functional grading was 0.82, both indicating excellent inter-observer agreement. Any inconsistent measurement or grading results generated by the 2 researchers were resolved through joint rereading and consensus discussion to reach a unified conclusion.

Preoperative fracture displacement was defined as the maximum separation distance of fracture fragments measured on anteroposterior and lateral elbow radiographs via built-in digital measurement software of the DR system, recorded in millimeters. The standardized Flynn elbow functional grading criteria covering elbow flexion range, extension loss, carrying angle deformity, and daily living activity recovery were adopted for postoperative functional evaluation at each follow-up visit.

### 2.4. Statistical analysis

Sample size calculation was performed prior to retrospective data collection. Based on previous domestic clinical cohort data on pediatric lateral condylar humeral fractures, we hypothesized a 15% difference in the excellent rate of Flynn elbow functional outcomes between the 2 groups. Setting α = 0.05, statistical power = 0.8, and a 1:1 group allocation ratio, the minimum required sample size for each group was calculated as 42 patients. Considering an estimated 10% loss to follow-up during the study period, we planned to enroll at least 47 subjects per group. Finally, 45 patients in Group A and 51 patients in Group B were enrolled in the present study, which basically satisfied the pre-calculated sample size requirement.

All statistical analyses were carried out using SPSS 26.0 software (IBM). The Shapiro–Wilk test was adopted to verify the normal distribution of continuous variables. Normally distributed continuous measurement data (age, operation duration, intraoperative blood loss, fracture healing time, elbow functional recovery time) were compared via independent samples *t*-test. Categorical enumeration data (gender, injured side, Flynn functional grading, complication incidence) were analyzed using the chi-square (χ^2^) test. *P* < .05 was defined as the threshold for a statistically significant difference.

## 3. Results

There were no differences between the 2 groups in preoperative variables (age, sex, lateral injury, and preoperative displacement), intraoperative variables (operative time and intraoperative bleeding volume), and postoperative variables (plaster fixation time, postoperative displacement, healing time, and recovery time of elbow function (*P* > .05; Table 1). All 96 patients achieved fracture healing and had no complications such as wound infection, nonunion, osteonecrosis, elbow varus, or valgus, and no differences were found in any of these groups (Tables 1 and 2).

**Table 1:** Demographic data and baseline clinical characteristics of the 2 groups.

Variables	Group A (n = 45)	Group B (n = 51)	Statistic	*P* value
Age	5.91 ± 2.18	6.02 ± 2.23	*t* = −0.240	.811
Sex			χ^2^ = 0.256	.613
Male	33	35		
Female	12	16		
Side injured			χ^2^ = 0.376	.539
Left	21	27		
Right	24	24		
Operation time	54.44 ± 11.45	53.25 ± 10.59	*t* = 0.529	.598
Intraoperative bleeding	21.93 ± 5.69	22.35 ± 6.28	*t* = −0.341	.734
Plaster fixation time	6.09 ± 0.67	6.20 ± 0.49	*t* = −0.705	.380
Postoperative displacement	0	0	–	–
Healing time	6.29 ± 0.73	6.08 ± 0.69	*t* = 1.171	.185
Time to recovery of elbow function	15.91 ± 4.00	15.73 ± 3.69	*t* = 0.236	.814
Infection	0	0	–	–
Nonunion	0	0	–	–

**Table 2:** Flynn functional outcome grading at final follow-up.

Flynn functional outcome grading	Group A (n = 45)	Group B (n = 51)	χ^2^	*P* value
Excellent	40 (88.9%)	42 (82.4%)	0.820	.365
Good	5 (11.1%)	9 (17.6%)		
Fair	0 (0.0%)	0 (0.0%)		

The Flynn functional outcome grading showed that the number of patients with excellent and good recovery was not significantly different between groups A and B (*P* = .365). None of the patients in either group had fair or poor functional outcomes.

## 4. Discussion

LCFH is an intra-articular fracture, and, like other intra-articular fractures, poor reduction can cause subluxation of the elbow, resulting in traumatic arthritis. Obtaining anatomical alignment is a fundamental requirement for treatment, and the treatment modalities of LCFHs are generally divided into the use of closed reduction and percutaneous pinning (CRPP) and ORIF. CRPP is recommended for fractures displaced <2 mm, and ORIF is recommended for fractures >2 mm.^[6-8]^ At present, there are also reports of large displaced LCFH with the use of CRPP,^[9]^ and arthroscopic assisted internal fixation,^[10]^ which received good results. Because closed reduction does not require extensive exposure, it can not only reduce further damage to the soft tissue and reduce blood loss. At the same time, it can greatly shorten the time required to expose the fracture end and to close the incision. Theoretically, it can also reduce the occurrence of complications such as nonunion, ischemic osteonecrosis, epiphyseal premature closure, and joint stiffness.^[11]^ And the postoperative elbow function recovery is faster, especially for fractures <4 mm. However, these closed reduction techniques are time-consuming, have high technical requirements, and have not been widely promoted in clinical practice.

Some scholars suggest that the closed reduction of LCFH should be tried first, but ORIF is still the gold standard.^[7,12]^. It has also been reported in the literature that the surgical method is not the major determinant of elbow functional recovery, and that fracture severity and classification are also important influencing factors.^[13]^ At present, ORIF remain the main treatments for LCFH. KW is a commonly used internal fixation material for LCFH in children, with simple operation and reliable fixation effect. K-wires <2 mm in diameter have a lower risk of damaging the epiphysis^[14]^; However, KWs are metal fixators, and leaving them outside the skin can easily lead to needle tract infection, osteomyelitis, and other complications.^[15]^ If left in the skin, an operation is necessary to remove the K-wire again after fracture healing, which increases the physical injury and psychological burden of the child and increases the risk of reanesthesia and the economic burden. With the increasing expectations of parents, avoiding the postoperative complications of LCFH in children and reducing surgical trauma is a problem that clinicians need to face.

The application of biodegradable pins is now recognized by an increasing number of clinicians and patients. Open reduction of biodegradable pins to fix larger displaced lateral condyle fractures avoids the pain caused by secondary removal of Kirschner pins in children. Open reduction with biodegradable pin fixation is easier to promote and recognize, and the results are reliable. Biodegradable pins have the following advantages: they have good biocompatibility and can be degraded in vivo without producing a rejection reaction, thus avoiding a second operation. Their strength can meet the fixation requirements for non-weight-bearing bone fractures. Early functional exercise can be performed, which is conducive to the recovery of elbow joint function. They avoid the anesthetic risks and pain associated with reoperation. The avoidance of a second operation is being accepted by more and more parents of children. Because the hardness and strength of biodegradable pins are significantly smaller than those of metal K-wires, some operators have concerns about their ability to stabilize external condyle fractures of the metal K-wire, some operators will have concerns that it is difficult to stabilize the external condyle fracture of the humerus. Therefore, some surgeons will assist in reinforcing the absorbable line tension band. This study aims to retrospectively analyze the necessity of auxiliary absorbable line tension bands. Although the strength of biodegradable pins is significantly lower than that of KWs, their 3-space distributed fixation provides adequate stability for pediatric lateral humeral condylar fractures.^[16]^ Recent biomechanical studies have demonstrated that the use of 3 biodegradable pins for 3-dimensional multi-planar fixation in pediatric lateral epicondylar fractures can provide adequate stability to the intra-articular fracture fragments.^[17]^ In recent years, multiple single-center retrospective cohort studies have confirmed the safety and efficacy of biodegradable implants in treating distal humeral intra-articular fractures in children.^[18,19]^ Findings from this study are consistent with those of previously published studies, with all subjects achieving complete fracture healing and no necessity for the use of absorbable tension band wiring observed. The greatest significance of this study lies in providing an evidence-based reference for the simplified clinical surgery of lateral humeral condyle fractures in children. There are some limitations. First, the follow-up time was too short, and the number of patients in both groups was small. Multi-center prospective randomized controlled trials with larger sample volumes are required to further validate the present conclusions. Second, this study only included Weiss type III fractures, and the conclusion cannot be directly extended to Weiss type I/II mildly displaced fractures. Future research can stratify fracture severity and conduct subgroup analysis to form more comprehensive clinical guidance. Third, the long-term effects of the absorbable line tension band on the epiphysis of the humeral condyle physis could not be evaluated.

## 5. Conclusion

The open reduction of biodegradable pins for the fixation of LCFH yielded satisfactory results without additional absorbable line tension band reinforcement.

## Author Contributions

**Data curation:** Dingding Jia.

**Formal analysis:** Zhenyu Chen.

**Investigation:** Liyuan Hao.

**Supervision:** Xiuyu Fan, Na Li.

**Writing – original draft:** Lifei Dong.

**Writing – review & editing:** Lifei Dong.
